# Robotic versus Laparoscopic Cholecystectomy: Case-Control Outcome Analysis and Surgical Resident Training Implications

**DOI:** 10.7759/cureus.7641

**Published:** 2020-04-11

**Authors:** Maher Ghanem, Samuel Shaheen, John Blebea, Faiz Tuma, Majd Zayout, Nico Conti, Ghaith Qudah, Mohamed K Kamel

**Affiliations:** 1 General Surgery, Central Michigan University College of Medicine, Saginaw, USA; 2 Surgery, Royal College of Surgeons in Ireland, Dublin, IRL; 3 Internal Medicine, University Hospitals Cleveland Medical Center, Cleveland, USA

**Keywords:** cholecystectomy, laparoscopic, robotic, clinical outcomes, surgical training

## Abstract

Background

The robotic approach in surgery is becoming more widely used in many subspecialties. Robot-assisted laparoscopic procedures provide potential improvements in clinical outcomes due to improved visualization and enhanced surgical ergonomics. In this study, we measured and compared outcomes of robot-assisted laparoscopic cholecystectomy with the conventional laparoscopic technique, as well as the implications for the training of surgical residents.

Method

We compared a total of 244 patients undergoing minimally invasive cholecystectomies performed by one surgeon between July 2013 and June 2016 examining relevant clinical outcomes including operative room (OR) time, length of hospital stay (LOS), readmission to the hospital, post-operative emergency department (ED) visits, and post-operative pain between laparoscopic single-incision cholecystectomy and robot-assisted laparoscopic cholecystectomy. A chi-square test and Student’s t-test were used to compare these variables between the two groups. Propensity score matching (PSM) was used using gender, age, and body mass index (BMI) as variables.

Results

From the total number of procedures of 244, 144 were included in the laparoscopic group and 100 in the robot-assisted group. The robot-assisted patients had a shorter post-operative LOS (mean: 0.8 vs. 1.6 days; p = 0.002). There was no significant difference in the OR time (mean: 64.8 vs. 65.0 minutes; p = 0.945), readmissions (4.0% vs. 3.5%; p = 0.830), post-operative ED visits (7.0% vs. 7.6%; p = 0.851), or post-operative pain (13.0% vs. 21.3%; p= 0.137). Robotic cholecystectomy patients were younger (mean: 46 vs. 52 years; p = 0.023) and had lower BMIs (mean: 31 vs. 33; p = 0.038). Because of these differences, we compared the two groups using PSM that confirmed the shorter LOS in the robotic group (mean: 0.9 vs. 1.9; p = 0.009).

Conclusions

These results demonstrate that robotic cholecystectomies can reduce LOS for patients undergoing laparoscopic cholecystectomy, without increasing OR time. Increased surgeon experience with robotic procedures and improved OR efficiency will allow greater opportunities for resident participation. Robotic training curricula need to be employed and objectively evaluated to improve surgical resident skill acquisition and provide earlier and progressive clinical participation in robotic procedures.

## Introduction

The robot-assisted approach is rapidly increasing in the performance of minimally invasive procedures in many surgical specialties including general surgery. As the robotic approach became more familiar to surgeons, progressively more procedures are being performed in this manner. Proponents of robotic surgery suggest that it provides better clinical outcomes than the conventional laparoscopic approach. This is based on factors such as improved dexterity provided by articulating instruments, augmented visualization with the implementation of three-dimensional viewing, improved stability and elimination of physiologic tremors, better cutting capabilities, and enhanced ergonomics, allowing for difficult surgical tasks to be performed safely with the use of smaller incisions [1,2].

Initial clinical studies of robot-assisted procedures focused on practicality and safety. There was less emphasis on other aspects of the clinical outcomes. Certain robotic procedures have already been shown to have better operative outcomes compared with open surgery in terms of length of hospital stay (LOS), complications and mortality, yet many studies comparing laparoscopic with robot-assisted surgeries have produced conflicting results in terms of clinical outcomes and cost-effectiveness [3-6]. Cholecystectomy is one of the most common procedures where the robot-assisted approach has been used. However, there are relatively few studies that compared clinical outcomes between laparoscopic cholecystectomy (LC) and robotic cholecystectomy (RC) [7].

With the current literature presenting mixed results of robot-assisted laparoscopic procedures, we reviewed our experience with RC and compared it with conventional LC in order to identify potential clinical benefits for this common surgical operation.

## Materials and methods

After obtaining approval from the university and hospital Institutional Review Boards, the clinical records of all patients operated upon by a single surgeon (M.G.) for cholecystectomy during a three-year period from 2013 to 2016 were reviewed. All patients aged 18 years or older who underwent laparoscopic or RC were included. Cholecystectomies performed in combination with other operative procedures were excluded. The total number of patients was 244, of whom 144 underwent conventional laparoscopic single-incision cholecystectomy and 100 underwent robot-assisted single-incision cholecystectomy. Data of the following variables were collected: operative time (OR; skin incision to skin closure), LOS, post-operative emergency department (ED) visits, readmission to the hospital, and post-operative pain. Post-operative pain was defined as any complaint of pain reported subjectively by the patient on post-operative visit documentation and quantified using a Likert single-dimensional scale.

There were significant differences in relevant patient characteristics between the two groups, with the robotic patients being six years younger and with a lower body mass index (BMI) (Table 1).

**Table 1:** Patient Characteristics SD, standard deviation

	Laparoscopic ( n = 144)	Robot-assisted ( n = 100)	p-Value
Patient characteristics			
Female gender, % (n)	66% (94)	68% (67)	0.810
Age in years, mean ± SD (n)	52 ± 19 (142)	46 ± 17 (99)	0.023
Body mass index, mean ± SD (n)	32.7 ± 7.9 (128)	30.6 ± 7.0 (97)	0.038

Therefore, we used propensity score matching (PSM) to create matched groups for statistical comparisons (Table 2) [8].

**Table 2:** Patient Characteristics after Propensity Score Matching SD, standard deviation

	Laparoscopic (n = 71)	Robot-assisted (n = 71)	p-Value
Patient characteristics			
Female gender, % (n)	63% (45)	68% (48)	0.596
Age in years, mean ± SD (n)	52 ± 20 (71)	50 ± 17 (71)	0.588
Body mass index, mean ± SD (n)	31.0 ± 6.8 (71)	31.5 ± 7.3 (71)	0.649

We used binary logistic regression, with gender, age, and BMI as independent variables, to calculate the propensity score and predicted probability of having a robotic surgery. We matched each robotic surgery patient with an LC patient based on matching PSM predicted probabilities. The matching technique provided a total of 142 patients, with 71 in each group. After matching, we used chi-square and/or Fisher’s exact test, as appropriate, to determine differences in the categorical variables of gender, readmission, post-operative ED visit, and the presence of post-operative pain. Quantitative data are reported as mean ± standard deviation. Statistical analysis was performed using Student’s t-test to assess differences in continuous variables between the matched groups and a p-value of <0.05 as showing statistical significance.

## Results

A summary of the clinical outcomes of the two groups of patients before propensity matching is presented in Table 3.

**Table 3:** Clinical Outcomes SD, standard deviation

	Laparoscopic (n = 144)	Robot-assisted (n = 100)	p-Value
Outcomes			
Operative time (minutes), mean ± SD (n)	65 ± 24 (136)	65 ± 22 (96)	0.945
Length of stay (days), mean ± SD (n)	1.6 ± 2.2 (144)	0.8 ± 1.7 (100)	0.002
Readmission, % (n)	3.5% (5)	4.0% (4)	0.830
Post-operative emergency department visit, % (n)	7.6% (11)	7.0% (7)	0.851

Compared with conventional LC, RC was associated with a one-day shorter post-operative LOS (mean: 0.8 vs 1.6; p = 0.002). There were no significant differences seen in OR time, readmissions, post-operative ED visits, or the presence of post-operative pain. The mean number of opioid pills prescribed per person in the laparoscopic group was 31 pills and that in the robotic group was 15 pills. The most common type of opioid prescribed was hydrocodone/acetaminophen 7.5-325 mg followed by hydrocodone/acetaminophen 5-325 mg and then acetaminophen/codeine 300-30 mg. Clinical outcomes among the 142 patients following propensity matching are summarized in Table 4.

**Table 4:** Clinical Outcomes after Propensity Score Matching SD, standard deviation

	Laparoscopic (n = 71)	Robot-assisted (n = 71)	p-Value
Outcomes			
Operative time (minutes), mean ± SD (n)	66 ± 24 (68)	66 ± 24 (69)	0.922
Length of stay (days), mean ± SD (n)	1.9 ± 2.3 (71)	0.9 ± 1.9 (71)	0.009
Readmission, % (n)	4.2% (3)	5.6% (4)	0.698
Post-operative emergency department visit, % (n)	9.9% (7)	8.5% (6)	0.771

The advantageous difference in LOS was maintained with RC being associated with a one-day shorter post-operative LOS as compared with conventional LC (0.9 vs 1.9; p = 0.009). There were no significant differences in OR time, readmissions, post-operative ED visits, or post-operative pain between the two PSM matched groups.

## Discussion

Robotic surgery is an established and evolving technology. Many institutions across North America have been using robotic systems such as the da Vinci system to perform various surgical procedures over the past 20 years [9]. With its popularity, many studies have been carried out to evaluate the benefits and drawbacks of this technology [1]. The benefits of robotic surgery include the ability to use instrumentation with dexterity, better visualization of the surgical field with the use of a three-dimensional view, eliminating tremors, and improved accuracy of cutting. With its many potential benefits, the use of robotics would have eclipsed the use of laparoscopic and open procedures. However, studies have shown mixed results as to whether robotic surgery is superior to other approaches. The high installment cost, the need for additional training, and the potentially increased operative time are negative observed drawbacks of the robotic approach [10,11].

Data on robotic surgeries cost and clinical outcomes are procedure-specific; therefore, robot-assisted outcomes have not shown consistent benefits, whereas other authors support robot-assisted over standard laparoscopy for cholecystectomies [4]. Salman et al. reviewed a national database and showed that robotic surgery had significantly shorter lengths of stay than open surgery and had lower charges than laparoscopic and open surgery [7]. The study found that robotic surgery had a lower mortality rate than non-robotic surgeries per 10,000 procedures. The overall cost was considered, including LOS, and robotic surgery appeared to be cost-effective and as safe as non-robotic surgery except in cholecystectomy and esophagogastric procedures [5]. Compared with the results of Kim et al. in 2014, which showed that most reported robotic surgeries have a longer operation time, this study showed less estimated blood loss, shorter LOS, lower complication and conversion rates, and comparable oncologic outcomes of robotic procedures compared with laparoscopic or open surgery [12].

A recent study by Hagen et al. in 2018 showed similar early post-operative clinical results when comparing robotic single-site cholecystectomy versus multiport LC but found a higher rate of re-operations and higher perioperative and long-term costs with robotic single-site cholecystectomy [13]. A meta-analysis comparing robotic with traditional minimally invasive surgery completed in 2016 showed a decrease in blood loss and transfusion rates. However, both techniques resulted in a similar LOS and 30-day overall complication rate [1].

Our study focused on the use of robot-assisted LC over conventional laparoscopy. Robotic procedures were found to be superior in one domain, LOS, but no significant difference in OR time. This could be due to patient selection for the robotic procedure and associated decreased operating times, with less severe or complex cases being performed robotically. It may also be due to the proficiency of the surgeon who has similar efficiency when performing either procedure. This suggests that with appropriate training for surgeons and operating room (OR) staff, there will be negligible differences in operating times between robotic procedures and LCs. This can be true in a training environment where surgical residents are involved in the procedure [14,15].

The beneficial difference of one day in the LOS for the robot-assisted approach is difficult to predict. The reduced LOS could reduce the risk of infection and hospital resource usage and cost [16]. However, cholecystectomy is now routinely performed as a same-day surgery except for inpatients who are admitted for other reasons. This impacts their LOS. The clinical impact of this advantage depends on the number and percentage of cholecystectomy procedures performed on inpatients in a particular institution.

When comparing the robotic approach with the conventional laparoscopic approach, studies have demonstrated that there is a possibility of patient harm due to machine system errors, video or imaging problems, electrical arcing, sparking, or instrumentation difficulties [17]. Within these studies, there were no intra-operative technical complications and no significant differences for readmission or post-operative ED visits when comparing robotic-assisted LC with conventional LC. This suggests that robotic techniques for cholecystectomies have similar levels of safety as laparoscopic techniques.

The presence of post-operative pain was also evaluated in this study. The mean number of opioid pills prescribed per person was greater in the laparoscopic group (31 pills) as compared with the robotic group (15 pills). However, this probably reflects the temporal trends of prescribing fewer post-operative opioids following cholecystectomies, with more robotic procedures performed in the latter part of the study period.

Although our sample size is not large, the analysis involved a single surgeon, which eliminates the confounding variable of varying surgeon skill level. Patients were carefully selected when the robot was first used, contributing to the differences in BMI and age. However, when controlling for these variables through PSM matching, the robotic technique still demonstrated an improvement in reducing LOS. In the obese population, studies have shown that an RC is a safe approach. Mitko et al. showed that RC reduced LOS for obese patients with BMI above 30 kg/m2, with no increased mortality or post-operative complications, except for increased incidence of retained common bile duct stones [18]. However, readmission for abdominal pain was six times less likely in the robotic group [16].

Another argument that supports the use of the robot-assisted approach is the need for residents’ training on robotic surgery in a teaching environment. The progressive integration of the robotics in multiple surgical procedures mandates earlier training for residents in this technology. A robotic training curriculum is a necessity of every surgery training program [19]. There is a large variation in the current content and structure of resident robotic training curricula, but they all include simulation, didactic sessions, system components and setup, and hands-on practice. The validity of simulation training in teaching fundamental robotic surgical skills and shortening the learning curve has been supported by major simulation companies such as Simulated Surgical Systems, Mimic Technologies Inc., Intuitive Surgical, and 3D Systems [19]. However, these curricula, unlike the standardized Fundamentals of Laparoscopic Surgery course, have not been formally evaluated. Chen et al. suggested comparing the surgical training efficacy among these varying curricula to develop a standardized robot-assisted surgical curriculum for educational and credentialing purposes [19]. Virtual simulation is available for robotic training, which could also be beneficial.

An important question facing teaching institutions is how to balance OR time with adequate training of surgery residents in robotic surgery [20]. Longer operative time while teaching residents on the robot has been demonstrated by more than one study [21,22]. This is especially true when there is only one console for both the attending surgeon and the resident trainee. The delay resulting from switching roles and the longer time needed by the resident to perform tasks are significant and may impede the opportunities for the residence to develop their skills. Discussion and selection of the appropriate intra-operative tasks between the attending surgeon and the resident, determined before the start of the procedure and appropriate to the training level and individual skills, is a reasonable approach to overcome this challenge.

Robotic hands-on practice with two consoles is very convenient and practical, even more than what is possible with laparoscopic training. Shifting between the surgeon and residents’ roles and guidance of the residents with marking pointers on the same operative screen are much more efficient than switching positions and instruments in the laparoscopic approach. Using a second console allows more opportunities for attending surgeons to facilitate training and teaching more difficult operative tasks to trainees. Such options, added to the additional enabling features of the robotic platform, suggest that residents are more likely to progress faster in robotic surgery than in traditional laparoscopic training.

Another relevant training consideration is the transferability of skills between the robotic and laparoscopic approaches. Both sets of skills should be taught as the residency programs need to prepare graduating residents for real-life practice. Current practice and the need for robotic and laparoscopic skills are continuously and unpredictably evolving. There is a significant spectrum of technical skills that overlap between the two approaches. This overlap has not been adequately studied nor quantified. Programs usually start training using the available training opportunities in the laparoscopic arena and then transfer these skills in the robotic approach. However, we believe that training in robotic skills should begin in the first year of residency rather than reserving it to the senior years of training. Both the availability of robotic simulators with the teaching consoles and the increasing numbers of general surgical procedures being performed laparoscopically indicate that junior residents should start this training at the beginning of their training and become involved in clinical cases in a progressive manner.

## Conclusions

RCs as compared with LC can reduce LOS and potentially associated hospital costs, without increasing OR time. Increased surgeon experience with robotic procedures and improved OR efficiency will allow greater opportunities for resident participation. Robotic training curricula need to be employed and objectively evaluated to improve surgical resident skill acquisition and provide earlier and progressive clinical participation in robotic procedures.

## References

[1] Tan A, Ashrafian H, Scott AJ, Mason SE, Harling L, Athanasiou T, Darzi A (2016). Robotic surgery: disruptive innovation or unfulfilled promise? A systematic review and meta-analysis of the first 30 years. Surg Endosc.

[2] Huang Y, Chua TC, Maddern GJ, Samra JS (2017). Robotic cholecystectomy versus conventional laparoscopic cholecystectomy: a meta-analysis. Surgery.

[3] Breitenstein S, Nocito A, Puhan M, Held U (2008). Robotic-assisted versus laparoscopic cholecystectomy: outcome and cost analyses of a case-matched control study. Ann Surg.

[4] Kamiński JP, Bueltmann KW, Rudnicki MJ (2014). Robotic versus laparoscopic cholecystectomy inpatient analysis: does the end justify the means?. Gastrointest Surg.

[5] Salman M, Bell T, Martin J, Bhuva K, Grim R, Ahuja V (2013). Use, cost, complications, and mortality of robotic versus non robotic general surgery procedures based on a nationwide database. Am Surg.

[6] Buzad FA, Corne LM, Brown TC (2013). Single-site robotic cholecystectomy: efficiency and cost analysis. Int J Med Robot.

[7] Ayloo S, Roh Y, Choudhury N (2014). Laparoscopic versus robot-assisted cholecystectomy: a retrospective cohort study. Int J Surg.

[8] Rosenbaum PR, Rubin DB (1983). The central role of the propensity score in observational studies for causal effects. Biometrika.

[9] Warren H, Dasgupta P (2017). The future of robotics. Investig Clin Urol.

[10] Trastulli S, Cirocchi R, Desiderio J (2020). Robotic versus laparoscopic approach in colonic resections for cancer and benign diseases: a systematic review and meta-analysis. PLoS One.

[11] Al Bandar MH, Al Sabilah J, Kim NK (2015). The current scope of robotic surgery in colorectal cancer. Adv Robot Autom.

[12] Kim CW, Kim CH, Baik SH (2014). Outcomes of robotic-assisted colorectal surgery compared with laparoscopic and open surgery: a systematic review. J Gastrointest Surg.

[13] Hagen M, Balaphas A, Podetta M (2018). Robotic single-site versus multiport laparoscopic cholecystectomy: a case-matched analysis of short- and long-term costs. Surg Endosc.

[14] George LC, O’Neill R, Merchant AM (2018). Residency training in robotic general surgery: a survey of program directors. Minim Invasive Surg.

[15] Ruurda JP, Broeders IA, Simmermacher RP, Borel Rinkes IH, Van Vroonhoven TJ (2002). Feasibility of robot-assisted laparoscopic surgery: an evaluation of 35 robot-assisted laparoscopic cholecystectomies. Surg Laparosc Endosc Percutan Tech.

[16] Rahmqvist M, Samuelsson A, Bastami S, Rutberg H (2016). Direct health care costs and length of hospital stay related to health care-acquired infections in adult patients based on point prevalence measurements. Am J Infect Control.

[17] Alemzadeh Alemzadeh, H H, Raman J, Leveson N, Kalbarczyk Kalbarczyk, Z Z, Iyer RK (2020). adverse events in robotic surgery: a retrospective study of 14 years of FDA data. PLoS One.

[18] Mitko J, Main W, Hussain L, Meister K, Kerlakian G, Tymitz K (2016). Laparoscopic versus robotic cholecystectomy in the obese population: is there a preferred approach?. Surg Obes Relat Dis.

[19] Chen R, Rodrigues Armijo P, Krause C, Siu KC, Oleynikov D (2020). A comprehensive review of robotic surgery curriculum and training for residents, fellows, and postgraduate surgical education. Surg Endosc.

[20] Honaker MD, Paton BL, Stefanidis D, Schiffern LM (2015). Can robotic surgery be done efficiently while training residents?. J Surg Educ.

[21] Nio D, Bemelman WA, Busch OR, Vrouenraets BC, Gouma DJ (2004). Robotic-assisted laparoscopic cholecystectomy vs conventional laparoscopic cholecystectomy. Surg Endosc.

[22] Delaney CP, Lynch AC, Senagore AJ, Fazio VW (2003). Comparison of robotically performed and traditional laparoscopic colorectal surgery. Dis Colon Rectum.

